# Protective Effects of Baicalin on Experimental Myocardial Infarction in Rats

**DOI:** 10.21470/1678-9741-2018-0059

**Published:** 2018

**Authors:** Longfei Wang, Yong Li, Shenglan Lin, Zhiqiang Pu, Haiping Li, Zhili Tang

**Affiliations:** 1 Department of Pharmacy, Nanchong Central Hospital, Nanchong, China.; 2 High School Biology Group, Nanchong Senior High School, Nanchong, China.; 3 Department of Pharmacy, General Hospital of Chengdu Military Region, Chengdu, China.

**Keywords:** Myocardial Infarction, Oxidative Stress, Bcl-2-Associated x Protein, Flavonoids

## Abstract

**Objective:**

This study aimed to investigate the protective effects of baicalin on myocardial infarction in rats and explore the related mechanisms.

**Methods:**

Fifty Sprague Dawley rats were randomly divided into the control, model, and low-, medium- and high-dose baicalin groups. The latter 3 groups were intraperitoneally injected with baicalin, with a dose of 12.5, 25 and 50 mg/kg, respectively. Then, the myocardial infarction model was established. The hemodynamic of rats was tested, the serum lactate dehydrogenase (LDH), creatine kinase-MB (CK-MB), prostacyclin (PGI_2_) and thromboxane A_2_ (TXA_2_) were determined, the myocardial superoxide dismutase (SOD) and malondialdehyde (MDA) levels were detected, and the myocardial B-cell lymphoma-2 (Bcl-2) and Bcl-2 associated X (Bax) protein expressions were determined.

**Results:**

Compared with the model group, in the high-dose baicalin group the ST segment height and LVEDP were significantly decreased (*P*<0.05), the LVSP was significantly increased (*P*<0.05), the serum LDH, CK-MB and TXA2 levels were significantly decreased (*P*<0.05), the PGI_2_ level was significantly increased (*P*<0.05), the myocardial SOD level was significantly increased (*P*<0.05), and the myocardial MDA level was significantly decreased (*P*<0.05); the myocardial Bcl-2 protein level was significantly increased, and the Bax protein level was significantly decreased (*P*<0.05).

**Conclusion:**

Baicalin has protective effects on myocardial infarction in rats. The possible mechanisms may be related to its resistance to oxidative stress, and up-regulation of Bcl-2 protein expression and down-regulation of Bax protein expression in myocardial tissue.

**Table:** 

Abbreviations, acronyms & symbols	
Bax	= Bcl-2 associated X
Bcl-2	= B-cell lymphoma-2
CK-MB	= Creatine kinase-MB
ELISA	= Enzyme-linked immunosorbent assay
HPLC	= High performance liquid chromatography
LDH	= Serum lactate dehydrogenase
LVEDP	= Left ventricular end diastolic pressure
LVSP	= Left ventricular systolic pressure
MDA	= Malondialdehyde
PGI_2_	= Prostacyclin
SD	= Sprague Dawley
SOD	= Superoxide dismutase
TXA_2_	= Thromboxane A_2_

## INTRODUCTION

Cardiovascular diseases are the major factors that threaten the people's health. They mainly refer to the function disorders in heart and vascular system, including hypertension, coronary heart disease, congestive heart failure, stroke and congenital heart disease. The ischemic heart disease is a major etiology of cardiovascular diseases, in which the coronary atherosclerotic heart disease occupies a large proportion^[1]^. Myocardial infarction is the most common cause of ischemic heart disease. It is mainly caused by myocardial ischemia due to coronary circulation disorder. Myocardial infarction is the common cause of death from cardiovascular diseases^[2]^. In addition, the incidence of arrhythmias caused by myocardial infarction is very high, with the high mortality rate^[3]^. *Radix Scutellariae* is the dried root of *Scutellaria baicalensis Georgi*. It is one of the commonly used Chinese medicinal herbs in the Asia region and has a long history of clinical application. Baicalin belongs to flavonoids. It is the main active component of *Radix Scutellariae* ^[4]^. Baicalin has a wide range of pharmacological effects, which are mainly presented in the antioxidant, free radical scavenging, anti-inflammatory, anti-tumor, blocking calcium channel, inhibiting aldose reductase, antiviral and anti-allergic aspects^[5-8]^. In addition, baicalin has protective effects on the immune, cardiovascular, digestive and nervous system^[9-11]^. This study investigated the protective effect of baicalin on experimental myocardial infarction in rats and explored the related mechanisms. The objective was to provide a theoretical basis for the development of baicalin related medicines for mitigation and treatment of myocardial infarction.

## METHODS

### Animal Grouping and Treatment

Fifty Sprague Dawley (SD) rats (200±20 g; Laboratory Animal Center of Sichuan University, Chengdu, China) were randomly divided into 5 groups: control group, model group, and low-, middle- and high-dose baicalin groups, 10 rats in each group. The rats in low-, middle- and high-dose baicalin groups were intraperitoneally injected with baicalin [High performance liquid chromatography (HPLC) purity ≥ 98%; Shanghai Jingdu Biological Technology Co., Ltd., Shanghai, China], with a dose of 12.5, 25 and 50 mg/kg, respectively. The rats in the control and in the model group were intraperitoneally injected with normal saline. The injection was performed once per day and was continued for 10 days. On the 8^th^ day, the rats in the model group, low-, middle- and high-dose baicalin groups were subcutaneously injected with isoprenaline (Shanghai Hefeng Pharmaceutical Co., Ltd., Shanghai, China) (20 mg/kg) once per day, for continued 2 days, thus the rat model of myocardial infarction was established. The rats in the control group were subcutaneously injected with the equivalent volume of normal saline. Finally, the electrocardiography was performed, and the value of ST-segment elevation was used as the index to assess the myocardial ischemia.

### Hemodynamic Test

At the 12 hours after the second injection of isoprenaline, a polyethylene plastic pipe with the diameter of 1 mm was inserted into the left ventricle of rats through the left carotid artery to perform the cardiac catheterization and was connected with the biological signal acquisition system. The left ventricular systolic pressure (LVSP) and left ventricular end diastolic pressure (LVEDP) were measured, which were used to evaluate the hemodynamic of rats.

### Determination of Serum Lactate Dehydrogenase, Creatine Kinase-MB, Prostacyclin and Thromboxane A_2_

Rats were anesthetized using sodium pentobarbital by intraperitoneal injection. The abdominal cavity was opened. The blood of the abdominal aorta was taken, followed by centrifugation at 256 X g for 10 min. The serum lactate dehydrogenase (LDH) and creatine kinase-MB (CK-MB) were detected using the enzyme-linked immunosorbent assay (ELISA). The serum prostacyclin (PGI_2_) and thromboxane A_2_ (TXA_2_) levels were determined using radioimmunoassay. The procedures were in accordance with the instructions of kits (Sigma-Aldrich Corp., MO, USA).

### Determination of Myocardial Superoxide Dismutase and Malondialdehyde

Heart of rats was taken, immediately followed by rinsing with saline. The 10% myocardial homogenate was made from 100 mg myocardial tissue using 5 ml of Tris-HCl solution (pH 7.4). After centrifugation at 626 X g for 10 min, the supernatant was obtained. The superoxide dismutase (SOD) and malondialdehyde (MDA) levels were determined by ELISA. The procedures were in accordance with the instructions of kits (Sigma-Aldrich Corp., MO, USA).

### Determination of Myocardial B-Cell Lymphoma-2 and Bcl-2 Associated X Protein Expression

The myocardial tissue of rats was homogenized and the protein was extracted. The expressions levels of B-cell lymphoma-2 (Bcl-2) and Bcl-2 associated X (Bax) protein were determined using Western blot assays. The procedures were in accordance with the instructions of kits (Fuzhou Maixin Biotechnology Development Co., Ltd., Fuzhou, China).

### Statistical Analysis

Statistical analysis was carried out using SPSS 22.0 software (SPSS Inc., Chicago, IL, USA). The data were presented as mean ± SD and were compared using single-factor analysis of variance test with SNK-q test. *P*<0.05 was considered statistically significant.

## RESULTS

### Effects of Baicalin on ST Segment Height in Rats

At the 12 hours after myocardial infarction modeling, the ST segment height in the control group, model group, and low-, middle- and high-dose baicalin groups was 0.15±0.03, 0.31±0.06, 0.28±0.04, 0.23±0.03 and 0.19±0.02 mV, respectively. The ST segment height in the model group was significantly higher than that in the control group (*P*<0.05), and that in the middle- and high-dose baicalin groups was significantly lower than that in the model group, respectively (*P*<0.05). However, the ST segment height in the three baicalin groups was significantly higher than that in the control group, respectively (*P*<0.05).

### Effects of Baicalin on Hemodynamics of Rats

**Fig. 1:**
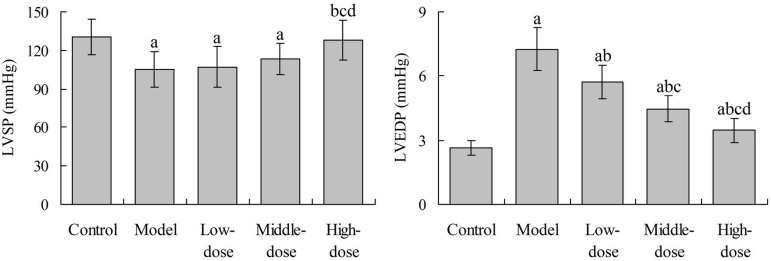
Effects of baicalin on hemodynamics of rats. ^a^P<0.05 compared with the control group; ^b^P<0.05 compared with the model group; ^c^P<0.05 compared with the low-dose group; ^d^P<0.05 compared with the middle-dose group. LVSP=left ventricular systolic pressure; LVEDP=left ventricular end diastolic pressure

Compared with the control group, the LVSP of rats in the model group was significantly decreased (*P*<0.05). Compared with the model group, the LVSP in the high-dose baicalin group was significantly increased (*P*<0.05), with no significant difference with the control group (*P*>0.05). Compared with the control group, the LVEDP in the model group were significantly increased (*P*<0.05). Compared with the model group, the LVEDP in the low-, middle- and high-dose baicalin groups was significantly decreased, respectively (*P*<0.05). However, the LVEDP in the three baicalin groups was significantly higher than that in the control group, respectively (*P*<0.05) (Figure 1).

### Effects of Baicalin on Serum LDH and CK-MB Levels in Rats

**Fig. 2:**
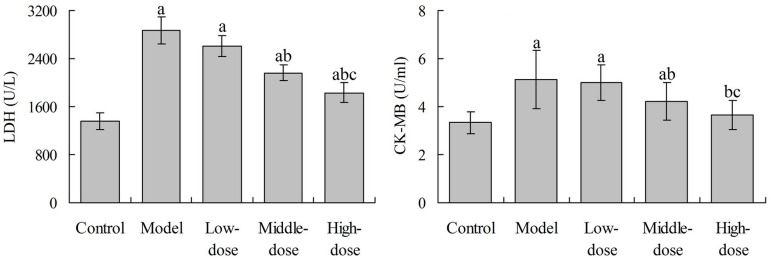
Effects of baicalin on serum LDH and CK-MB level in rats. ^a^P<0.05 compared with the control group; ^b^P<0.05 compared with the model group; ^c^P<0.05 compared with the low-dose group. LDH=lactate dehydrogenase; CK-MB= creatine kinase-MB

Serum LDH and CK-MB levels in rats in the model group were significantly increased when compared with the control group (*P*<0.05). Compared with the model group, the LDH and CK-MB levels in the middle- and high-dose baicalin groups were significantly decreased, respectively (*P*<0.05). However, the LDH level in the three baicalin groups and the CK-MB level in the low- and middle-dose baicalin group were significantly higher than those in the control group, respectively (*P*<0.05) (Figure 2).

### Effects of Baicalin on Serum PGI2 and TXA2 levels in Rats

**Fig. 3:**
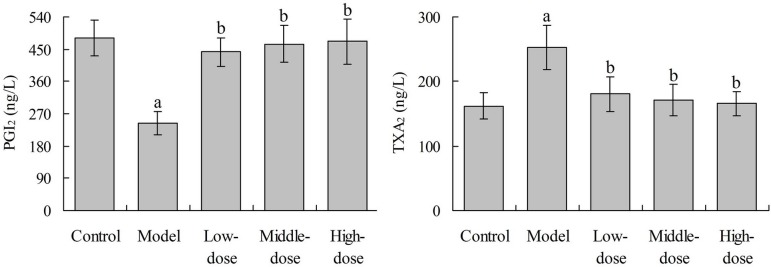
Effects of baicalin on serum PGI_2_ and TXA_2_ level in rats. ^a^P<0.05 compared with the control group; ^b^P<0.05 compared with the model group. PGI_2_=prostacyclin; TXA_2_=thromboxane A_2_

Compared with the control group, the serum PGI_2_ level in rats in the model group was significantly decreased (*P*<0.05). Compared with the model group, the PGI_2_ level in the three baicalin groups was significantly increased, respectively (*P*<0.05). Compared with the control group, the serum TXA_2_ level in the model group was significantly increased (*P*<0.05). Compared with the model group, the TXA_2_ level in the three baicalin groups was significantly decreased, respectively (*P*<0.05). There was no significant difference in each index between each baicalin group and control group (*P*>0.05) (Figure 3).

### Effects of Baicalin on Myocardial SOD and MDA in Rats

**Fig. 4:**
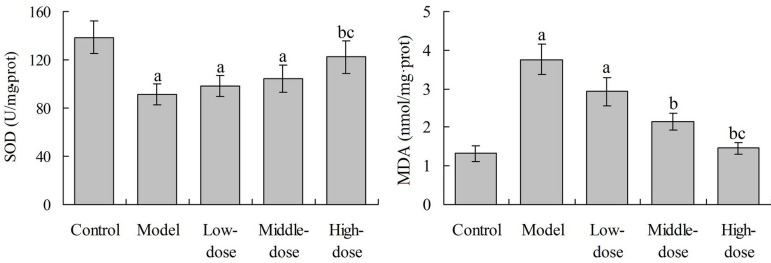
Effects of baicalin on myocardial SOD and MDA in rats. ^a^P<0.05 compared with the control group; ^b^P<0.05 compared with the model group; ^c^P<0.05 compared with the low-dose group. SOD=superoxide dismutase; MDA=malondialdehyde

As shown in Figure 4, compared with the control group, the myocardial SOD level in rats in the model group, low- and middle-dose baicalin groups was significantly decreased, respectively (*P*<0.05). Compared with the model group, the SOD level in the high-dose baicalin group was significantly increased (*P*<0.05). Compared with the control group, the myocardial MDA level in the model group and low-dose baicalin groups was significantly increased, respectively (*P*<0.05). Compared with the model group, the myocardial MDA level in the middle- and high-dose baicalin groups was significantly decreased, respectively (*P*<0.05).

### Effects of Baicalin on Myocardial Bcl-2 and Bax Protein Expression in Rats

Table 1 showed that, compared with the control group, the myocardial Bcl-2 protein level in rats in the model group, and low- and middle-dose baicalin groups was significantly decreased, respectively (*P*<0.05). Compared with the model group, the Bcl-2 protein level in the high-dose baicalin group was significantly increased (*P*<0.05). Compared with the control group, the myocardial Bax protein level in the model group, low- and middle-dose baicalin groups was significantly increased, respectively (*P*<0.05). Compared with the model group, the myocardial Bax protein level in the high-dose baicalin group was significantly decreased (*P*<0.05).

**Table 1:** Effects of baicalin on myocardial Bcl-2 and Bax protein expression in rats.

Group	n	Bcl-2/β-actin	Bax/β-actin
Control	10	1.45±0.12	0.35±0.03
Model	10	1.21±0.13^a^	0.84±0.06^a^
Low-dose	10	1.19±0.16^a^	0.92±0.09^a^
Middle-dose	10	1.24±0.11^a^	0.58±0.08^a^
High-dose	10	1.40±0.21^bcd^	0.43±0.06^bc^

a *P*<0.05 compared with the control group;

b *P*<0.05 compared with the model group;

c *P*<0.05 compared with the low-dose group;

d *P*<0.05 compared with the middle-dose group Bcl-2=B-cell lymphoma-2; Bax=Bcl-2 associated X protein

## DISCUSSION

It is found that the large-dose intravenous injection of isoprenaline can induce acute myocardial infarction, especially in the endocardium of the left ventricle and septum. The pathophysiological changes and myocardial morphological abnormalities induced by isoprenaline are similar to those of human myocardial infarction. Therefore, isoprenaline-induced myocardial infarction model is widely used to study the pathophysiological disturbances and morphological abnormalities of myocardial infarction and evaluate the effect of corresponding drugs^[12]^. Hemodynamic abnormalities often occur during myocardial infarction, with decreased cardiac diastolic and systolic function and increased myocardial oxygen consumption^[13]^. Results of this study showed that, after injection of with isoprenaline, the ST segment height and LVEDP in rats were significantly increased, respectively, and the LVSP was significantly decreased. This indicates that the myocardial infarction of rats has been successfully created. Compared with the model group, the ST segment height and LVEDP in groups with pretreatment using a certain dose of baicalin were significantly decreased, and the LVSP was significantly increased, respectively. This indicates that baicalin can improve the cardiac systolic and diastolic function, reduce the myocardial oxygen consumption, improve ventricular compliance, and mitigate the myocardial ischemia.

The activity of plasma LDH and CK-MB indirectly reflect the integrity of myocardial cell membrane and the degree of myocardial injury^[14]^. In myocardial cell necrosis, the integrity of myocardial cell membrane is damaged, with increased permeability, which results in the leakage of intracellular LDH and CK-MB into plasma, leading to the increased LDH and CK-MB concentration in blood^[15]^. Results of this study showed that the serum LDH and CK-MB levels in model group were significantly increased when compared with control group, respectively. Compared with the model group, the LDH and CK-MB levels in middle- and high-dose baicalin group were significantly decreased, respectively. This indicates that the pretreatment with baicalin can prevent the leakage of intracellular LDH and CK-MB into plasma, thus exerting the myocardial protection functions.

TXA_2_ can promote the platelet aggregation and the contraction of the coronary arteries^[16]^. On the contrary, PGI_2_ is known as the most potent inhibitor of platelet aggregation, with cytoprotective effect indirectly expanding coronary artery and inhibiting the production of oxygen free radicals^[17]^. In myocardial infarction, the arterial endothelial is damaged, so the PGI_2_ synthesis in coronary artery endothelial cells is decreased. Therefore, the platelet aggregates in the subendothelial collagen tissue, and excessively activates the production of a large number of TXA_2_^[18]^. In the present study, compared with the control group, the serum PGI_2_ level in rats in model group was significantly decreased, and the TXA_2_ level was significantly increased. Compared with the model group, the PGI_2_ level in the three baicalin groups was significantly increased, and the TXA_2_ level was significantly decreased. This suggests that baicalin can prevent the decrease of PGI_2_ level and the increase of TXA_2_ level in myocardial infarction rats, which may be related to its myocardial protective effects.

In isoprenaline-induced myocardial infarction model, the pathogenesis is related to lipid peroxidation, free radical production, membrane permeability changes and calcium overload in the myocardium^[19]^. SOD is an important antioxidant enzyme in the body. It can catalyze the transformation of oxygen free radicals to hydrogen peroxide, thus avoiding the damage to cells. MDA is one of the final products of cell membrane lipid peroxidation. It indirectly reflects the degree of cell membrane peroxidation^[20]^. Results of this study showed that, compared with the control group, the myocardial SOD level in the model group was significantly decreased, and the myocardial MDA level in the model group was significantly increased. Compared with the model group, the SOD level in the high-dose baicalin group was significantly increased, and the myocardial MDA level in the middle- and high-dose baicalin groups was significantly decreased, respectively. This suggests that baicalin has the ability of scavenging radical and reducing lipid peroxidation, thus playing a role in myocardial protection in rats.

Bcl-2 gene is a specific survival gene for inhibiting the apoptosis of cells. It plays an important role in the regulation of cell apoptosis^[21]^. Bcl-2 can resist various forms of cell death and prolong the life-span of cells, which leads to the increase of cells^[22]^. Bax gene is the apoptosis-promotion gene. Bax gene and Bcl-2 gene belong to the same gene family. Bax can not only inhibit the apoptosis inhibition effect of Bcl-2, but also directly promote the apoptosis of cells^[23]^. In this study, compared with the control group, the myocardial Bcl-2 protein level in the model group was significantly decreased, and the myocardial Bax protein level was significantly increased. Compared with the model group, the Bcl-2 protein level in the high-dose baicalin group was significantly increased, and the Bax protein level was significantly decreased (*P*<0.05). This indicates that baicalin can up-regulate the expression of the Bcl-2 protein and down-regulate the expression of the Bax protein in rats, thus mitigating the myocardial infarction.

## CONCLUSION

Baicalin has protective effects on myocardial infarction in rats. The possible mechanisms may be related to its resistance of oxidative stress, and up-regulation of the Bcl-2 protein expression and down-regulation of the Bax protein expression in myocardial tissue. This study has provided a theoretical basis for the development of baicalin related medicines for mitigation and treatment of myocardial infarction. This study still has some limitations. ST segment height, LVEDP and serum LDH in the baicalin groups still had significant differences with the control group (*P*<0.05). The reason may be that the baicalin doses used in this study are not enough to largely lower above indexes in the myocardial infarction rats to normal levels. Therefore, other suitable doses of baicalin should be further investigated. In addition, whether there are other mechanisms in the protective effects of baicalin on myocardial infarction needs to be further confirmed.

**Table:** 

Authors' roles & responsibilities	
LW	Agreement to be accountable for all aspects of the work in ensuring that questions related to the accuracy or integrity of any part of the work are appropriately investigated and resolved; final approval of the version to be published
YL	Agreement to be accountable for all aspects of the work in ensuring that questions related to the accuracy or integrity of any part of the work are appropriately investigated and resolved; final approval of the version to be published
SL	Agreement to be accountable for all aspects of the work in ensuring that questions related to the accuracy or integrity of any part of the work are appropriately investigated and resolved; final approval of the version to be published
ZP	Agreement to be accountable for all aspects of the work in ensuring that questions related to the accuracy or integrity of any part of the work are appropriately investigated and resolved; final approval of the version to be published
HL	Agreement to be accountable for all aspects of the work in ensuring that questions related to the accuracy or integrity of any part of the work are appropriately investigated and resolved; final approval of the version to be published
ZT	Substantial contributions to the conception or design of the work; or the acquisition, analysis, or interpretation of data for the work; drafting the work or revising it critically for important intellectual content; final approval of the version to be published
